# Ceria Particles as Efficient Dopant in the Electrodeposition of Zn-Co-CeO_2_ Composite Coatings with Enhanced Corrosion Resistance: The Effect of Current Density and Particle Concentration

**DOI:** 10.3390/molecules26154578

**Published:** 2021-07-29

**Authors:** Marija Riđošić, Mihael Bučko, Asier Salicio-Paz, Eva García-Lecina, Ljiljana S. Živković, Jelena B. Bajat

**Affiliations:** 1 Faculty of Technology and Metallurgy, University of Belgrade, Karnegijeva 4, 11000 Belgrade, Serbia; mridjosic@tmf.bg.ac.rs or; 2 Faculty of Technology Zvornik, University of East Sarajevo, Karakaj 34A, 75400 Zvornik, Republic of Srpska, Bosnia and Herzegovina; 3 Military Academy, University of Defense, Veljka Lukića Kurjaka St 33, 11000 Belgrade, Serbia; mbucko@tmf.bg.ac.rs; 4 CIDETEC Basque Research and Technology Alliance (BRTA), 20014 Donostia-San Sebastián, Spain; asalicio@cidetec.es (A.S.-P.); egarcia@cidetec.es (E.G.-L.); 5 Vinča Institute of Nuclear Science, University of Belgrade, 11000 Belgrade, Serbia; ljzivkovic@vin.bg.ac.rs

**Keywords:** composite coatings, electrodeposition, ceria powder, ceria sol, corrosion resistance, ultrasound

## Abstract

Novel Zn-Co-CeO_2_ protective composite coatings were deposited successfully from chloride plating solutions. Two different types of ceria sources were used and compared: commercial ceria powder and home-made ceria sol. Electrodeposition was performed by a direct current in the range of 1–8 A dm^−2^. Two different agitation modes were used and compared, magnetic stirring and ultrasound-assisted stirring (US). The influence of magnetic stirring on the stability of the related plating baths was evaluated via a dynamic scattering method. The results pointed to better stability of the prepared ceria sol. The morphology of the composite coatings was examined by scanning electron microscopy (SEM), and particle content was determined by energy-dispersive X-ray spectroscopy (EDS). The results showed that the increase in the deposition current density was not beneficial to the coating morphology and particle content. The corrosion behavior of the Zn-Co-CeO_2_ composite coatings was analyzed and compared by electrochemical impedance spectroscopy and polarization resistance. The ultrasound-assisted electrodeposition at small current densities was favorable for obtaining composite coatings with enhanced corrosion stability. The protection was more effective when US was applied and, additionally, upon utilization of ceria sol as a particle source, which was revealed by higher polarization resistance and greater low-frequency impedance modulus values for sol-derived composite coatings deposited under ultrasound.

## 1. Introduction

Metal matrix composites (MMC), formed by a metal matrix and particles with a different size and nature as a reinforcing material, are one of the most promising ways for enhancing the durability of sacrificial coatings deposited on steel [1], for instance, zinc coatings. A wide range of particles can be used in zinc composite coatings, such as TiO_2_ [2], SiC [3], Al_2_O_3_ [4], and ZrO_2_ [5], aiming to enhance the durability, corrosion protection, or hardness of the metal matrix. Zn alloy composites are also considered for enhancing the performance of such coatings, as the alloying of Zn with metals such as Mn [6,7], Ni [8,9], Fe [8,10], or Co [11,12] showed to be a good strategy for providing improved corrosion protection of steel.

The MMC can be produced by different routes, such as powder metallurgy [13], metal spraying [14], physical vapor deposition (PVD), chemical vapor deposition (CVD) [15], or nitridation [16]. One of the main drawbacks of these techniques, among others, is working at high temperatures. In this scenario, electrodeposition is a very good alternative for MMC production due to the use of affordable equipment, the possibility of working at room temperature, the ability to coat on complex shapes, and the easy transferring of the technology from laboratory to industrial scale [17]. 

However, the performance of MMC depends on the particle incorporation as well as on their distribution inside the matrix, which, on the other hand, depends on the working parameters of the electrodeposition process (i.e., current density, particle content in the bath, pH, and stirring) [16]. Thus, the influence of all these parameters has to be explored in order to optimize the performance of the MMC coating.

One of the main parameters ruling the amount of particles incorporated in the metal matrix is current density [16,17,18]. However, it is difficult to establish a direct relation between the deposition current density and particle content in the coating, as it can vary broadly depending on the selected particles. Usually, the particle content grows with an increase in the current density and particle loading in the bath until the saturation point is reached [16], but this is not always the case. The particle incorporation rate can decrease continuously or exhibit a maximum with a rise of current density, and such phenomena is related to metal deposition behavior [18]. Tuaweri et al. examined the influence of current density (1–5 A dm^−2^) and particle load (13–52 g dm^−3^) on SiO_2_ content in a Zn-Ni matrix and found irregular correlations [17]. The maximum particle content in the matrix was obtained at a 3 A dm^−2^ current density and 40 g dm^−3^ particle concentration in the bath. Ranganatha et al. showed that increasing CeO_2_ concentration in the plating bath enhanced the polarization resistance and reduced the roughness of Zn-CeO_2_ composite coating [19]. The rise in particle concentration in the plating bath from 5 to 10 g dm^−3^ caused the improvement in the incorporation rate of SiC into the Zn matrix. Conversely, a higher plating bath load (15 g dm^−3^) showed a drop in the particle incorporation rate due to the evident agglomeration phenomenon [20].

In MMC plating, a higher particle incorporation does not necessarily result in a better corrosion protection. For instance, the incorporation of SiC into the Zn matrix improved the dissolution of the matrix [20]. The incorporation of TiO_2_ particles and micro-hardness showed an initial enhancement by increasing the current density from 2 to 4 A dm^−2^, but a further increase to 8 A dm^−2^ provoked the decrease in both features [2]. A similar trend was reported by Guo at al [21], i.e., the maximum of the particle content was obtained at 8 A dm^−2^, but, with a further rise of the current density, the incorporation rate was slowed down and the coatings’ properties deteriorated. The increase in current density can also show a negative effect on incorporation rate (i.e., lack of maximum)as in the case of Zn-SiC composite coatings [3], where particle content decreased with the shift of current density from 8 to 12 A dm^−2^. The relationship between the incorporation rate and the current density could be related to a fast dissolution of the anode and a faster transport of metal ions toward the cathode, compared to the situation where particles are transported by agitation [3]. On the other hand, too low a current density is not beneficial to the particle incorporation rate. Saha and Khan [22] showed that 1 A dm^−2^ was more effective for Al_2_O_3_ incorporation compared to those obtained at 0.5, 2, and 3 A dm^−2^. At low current densities, the incorporation of particles was reduced due to the lower current efficiency [22]. The changes in particle incorporation rate with altering the deposition current density indicate that different factors, such as the rate of metal deposition on the cathode, the shape of particles, agitation, and the electrostatic interaction between charged particles and the cathode, play an important role in MMC plating.

All these findings suggest that the optimum current density and plating bath composition have to be determined in order to obtain a beneficial concentration of the particles in the deposit. We are not aware of any available literature on the influence of current density and bath loading on the properties of Zn-Co-CeO_2_ nanocomposite coatings. Thus, the main aim of this work is to obtain Zn-Co-CeO_2_ composite coatings and to optimize the electrodeposition parameters. The influence of the deposition current density and concentration of particles in the plating bath on the incorporation rate and corrosion resistance of the obtained composite coating was studiously examined. Two different sources of ceria particles were used, a commercial nanopowder and home-made sol. The ceria sol, as a stable dispersion of colloidal particles, was used in order to minimize the agglomeration rate of the particles, as nanoparticles tend to agglomerate in order to reduce their surface energy [23]. The agitation of the plating solution was provided either by a magnetic stirrer or an ultrasonic bath.

## 2. Experimental Part

The Zn-Co-CeO_2_ composite coatings were deposited from a plating solution containing 0.1 mol dm^−3^ ZnCl_2_; 0.8 mol dm^−3^ H_3_BO_3_; 3 mol dm^−3^ KCl and 0.03 mol dm^−3^ CoCl_2_·6H_2_O; and 2or 5 g dm^−3^ CeO_2_.The pH of the solution was fixed at 3 and the temperature at 24 °C in all the experiments. Chemicals were of analytical grade produced by Sigma-Aldrich (Saint Louis, MO, USA). Two different CeO_2_ sources were used during the experiments, commercial CeO_2_ nanopowder suspended in double-distilled water (Sigma Aldrich, spherical particles, size <50 nm) and an in-house synthesized stable aqueous colloidal dispersion (sol); all chemicals were dissolved in sol in the latter. The synthesis procedure of ceria sol is explained in detail elsewhere [24]. As in the case of the nanopowder, two concentrations of ceria sol (2 or 5 g dm^−3^) were studied. Sol of higher solid content was obtained by pressure filtration from the lower solid containing one. Ceria sol particles were spherical, exhibiting monomodal and narrow size distribution, with an average size of hydrodynamic diameters around 60 nm. The stability of sol particles was ensured by high positive surface charge (Z-potential cca 45 mV). Although composed of nanometric primary units, ceria powder was agglomerated in water and the dominant particle size was 1124 nm [23].

The size of particles in the electrolytic baths containing sol or powder ceria source was measured by a Zetasizer Nano ZS with 633 nm He-Ne laser (Malvern, UK). The hydrodynamic particle sizes were determined by the dynamic light scattering (DLS) method, in which results are given by intensity of the scattered light [25,26]. The Z-average particle size (Zav) or Z-average mean (also known as cumulants mean) is the primary parameter produced by this technique and the most commonly used DLS size parameter for quality control purposes. Polydispersity index (PDI) is a measure of the broadness of the size distribution calculated from the cumulant analysis and ranges from 0 to 1 (nearly monodisperse <0.1, moderate 0.1–0.7, and very broad distribution >0.7). Another first order result is an intensity distribution of particle sizes (PDS), which is of interest if the analyzed system shows a broader size distribution. The intensity is naturally weighted according to the scattering intensity of each particle fraction and is commonly given as Intensity % (share in intensity of the scattered light). For a perfectly monodisperse sample, the two results should be the same, i.e., the Zav should be the same as the mean of the (one and only) peak in the distribution. In real applications, even for monodisperse samples, this is likely not the case, and there will be small differences. For polydisperse samples, the two factors cannot be the same. The Z-average will still be only a single number, whereas the distribution will usually show two or more peaks or even only one [27]. Thus, it is useful to take into consideration both results for interpreting the data correctly. The duration of each PSD measurement was around 3 min. The composite coatings were deposited by direct current on a steel working electrode (AISI-1010, active area 8 cm^2^), while pure zinc was used as an anode. Two types of stirring were used and compared: a magnetic stirrer (300 rpm) and an ultrasonic bath (Bandelin electronics, 35 kHz, calculated power was 38.7 W cm^3^). The composite coatings were deposited at different current densities in the range 1–8A dm^−2^. The deposition time was initially calculated according to Faraday’s law to obtain ~10-μm-thick coatings. The deposit thickness was measured by the magnetic induction method, using Dualscope MPOR, and the current efficiency was calculated by comparing the thickness obtained and the thickness predicted by Faraday law. Subsequently, when it was necessary, the coatings were deposited with an adjusted time to obtain the targeted 10 μm thickness.

The morphology of the composite coatings was examined by scanning electron microscopy (SEM) (Tescan Mira), and composition was determined by energy-dispersive X-ray spectroscopy (EDAX) (Shimadzu EDX-8000). The corrosion resistance of the produced coatings was examined by linear polarization resistance and electrochemical impedance spectroscopy performed using Gamry Reference 600 Potentiostat. Electrochemical impedance tests were performed at open circuit potential, in the 100 kHz–0.01 Hz frequency range, with 10 mV amplitude and 7 points/decade. For linear polarization measurements, each sample (working electrode) was potentiodynamically polarized in the range between −15 and +15 mV (from cathodic to anodic) over the stabilized open circuit potential (OCP). At least three measurements were performed for each sample, with very good reproducibility.

## 3. Results and Discussion

### 3.1. Stability of Plating Solutions

**Figure 1:**
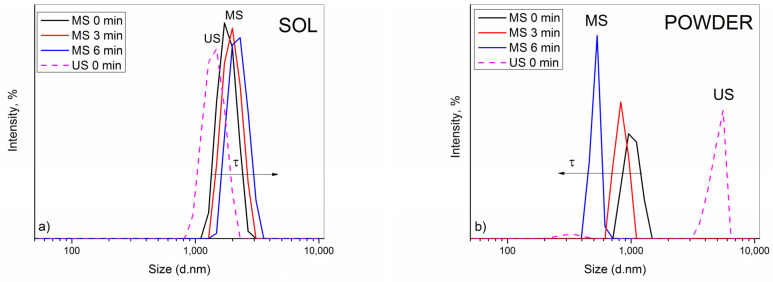
Time dependence of PSD of (**a**) ceria sol and (**b**) ceria powder-containing electrolytic baths after magnetic stirring (MS). PSD of analogous baths after ultrasound stirring (US) is given for comparison (dash lines) [23]. Ceria content was set at 2 g dm^−3^.

**Figure 2:**
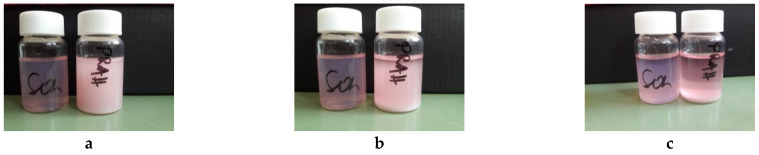
Visual observation of ceria sol (bottles on the left) and ceria powder-containing (bottles on the right) electrolytic baths after magnetic stirring: (**a**) 0 min, (**b**) 3 min, and (**c**) 6 min.

In this study, the influence of stirring mode on the stability of the composite baths was evaluated by acquiring the PSD data, as well as by visually monitoring the sedimentation behaviors at the same time intervals (Figure 1 and Figure 2). Three consecutive size measurements were collected for each system, enabling dynamic monitoring of the aggregation phenomena. Namely, the effect of the magnetic stirring was investigated in detail, and the findings were compared with those from our recent paper where application of US was thoroughly studied [23]. In parallel, the influence of the ceria particle source, sol or powder, at a solid content of 2 g dm^−3^ was followed. It was shown [23] that the incorporation of other constituents into ceria sol or ceria powder dispersion provoked destabilization of particles due to the increase in ionic strength of the plating solution.

In analogy with the US, similar phenomenon was recorded after application of MS.As seen in Figure 1a, the electrolytic bath containing sol exhibited nearly Gaussian-like PSD spectra. A continuous small growth in particle diameters with time indicated a relatively stable system with low PDI (≈0.250), suggesting that the aggregation process of particles occurred slowly. The average value of particle diameters, Zav, shifted accordingly with time, i.e., 1812, 1883, and 2098 nm. The most abundant particle fraction, situated at 1881, 1992, and 2231 nm, in due order, corresponded well to Zav, which confirms that this value actually reflects the real size of aggregates throughout the bath and points to their stability. Visual observations showed good agreement with PSD data, as the difference in transparency among the bottles with sol-containing bath was practically negligible. Only after 6 min the formation of small sediment was noticeable, indicating that particles started to aggregate and settle slowly. However, only lower ceria content sol was acceptable for deposition experiments. The plating bath based on 5 g dm^−3^ CeO_2_ was unstable and settled rapidly. As a result, the obtained coatings were not uniformly formed on the steel substrate; thus, this ceria sol was exempt from further consideration. It is likely that at a higher solid concentration, ceria sol, as a charge-stabilized colloid, reached a critical coagulation concentration (CCC) upon addition of other bath constituents. The CCC is the concentration of electrolyte necessary to bring the system into the regime of rapid coagulation instantaneously [28,29].

The PSD spectra of the ceria powder-containing bath, Figure 1b, showed the opposite size trend, however, only apparently. A continuous reduction in diameters of the most abundant particle size population, positioned at 1026, 833.4, and 515.0 nm, recorded in consecutive measurements, would incorrectly suggest an improvement in stability. A huge discrepancy with Zav value (1621, 1984, and 2254 nm) along with increasing PdI (0.412, 0.637, and 0.995) in due order, undoubtedly indicated the presence of much bigger particles in the bath and an enhanced polydispersity. In fact, the aggregation process occurred very fast, i.e., large-sized particle aggregates/clusters were formed quickly and sedimented rapidly, resulting in smaller and smaller sized particles that remained suspended in the bath and scattered the light. A visual observation of the glass bottles revealed that the bath became more clearer in time while thicker sediment was formed.

Based on the presented data, it can be concluded that the ceria sol provided greater stability compared to the ceria powder when the electrolytic bath was stirred magnetically, in analogy to the conclusion of our recently published article [23], where the difference in dispersion behavior between the electrolytic bath containing ceria sol and powder after the application of the US agitation was investigated. It was concluded that the sol was superior to the powder, imparting better stability.

However, it is obvious that MS was less efficient than US agitation, with regard to the stability imparted to the bath (Figure 1 dash lines). In the sol-containing bath, particles were of bigger diameters when subjected to MS compared to those measured after US (Figure 1a). The same conclusion stands for the powder-containing bath (Figure 1b). Application of US provided more efficient particle de-agglomeration, resulting in formation of similarly sized aggregates in the bath. The most abundant size population was at 4979 nm (94.9%), which reflected the measured Zav (4931 nm) well, pointing to the relative homogeneity and stability of the bath. Unlike after MS, where the particles aggregated and settled quickly, as already discussed, and only small-sized ones remained in the bath (Figure 2).

### 3.2. Deposition under Magnetic Stirring

#### 3.2.1. Cathodic Potentiodynamic Polarization Curves

**Figure 3:**
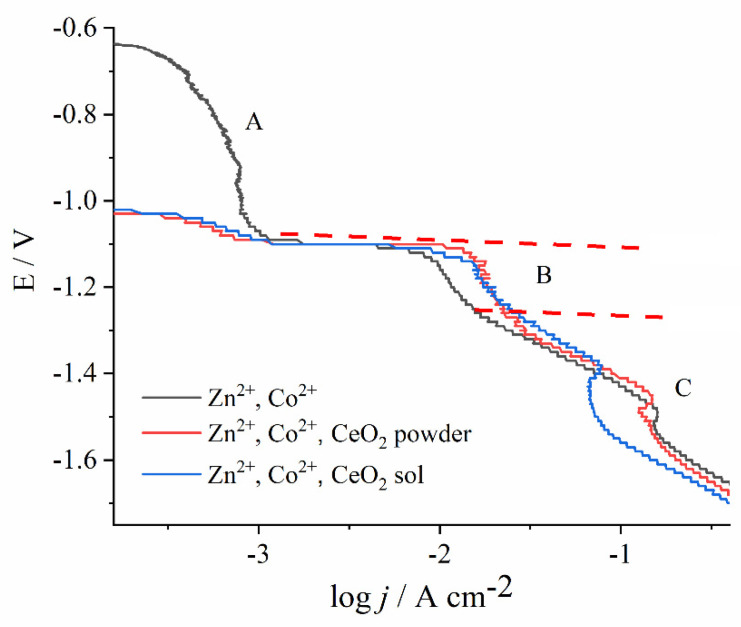
Effect of ceria present in the form of powder or sol in the Zn- and Co-containing bath on the cathodic potentiodynamic curves on steel substrate. The polarization is carried out with magnetic stirring at pH 3.00, with a scan rate of 1 mV s^−1^.

Figure 3 shows the potentiodynamic curves for solutions containing Zn^2+^ and Co^2+^ ions as well as with both types of CeO_2_ sources. The electrolytes were agitated with a magnetic stirrer, at a 300 rpm rate. The curves show the same shape, characteristic for the Zn-Co alloy electrodeposition, and can be divided into regions, according to previous research [30,31].

The region A is known as a normal deposition region, where only Co electrodeposition occurs. The suppressed current in this region evidences that the process is strongly inhibited. The suppression of Co^2+^ reduction was attributed to Zn underpotential deposition (UPD) on steel substrate but also on freshly formed Co nuclei [32]. At a sufficiently high Co^2+^ concentration, the current peak could have been observed [30,31], yet in our work, with only 0.03 mol dm^−3^ of Co^2+^, the Co^2+^ reduction was recorded as a weak current hump.

At the onset of region B, the sharp rise in current density denotes the Zn^2+^ electroreduction, coupled with the still suppressed Co^2+^ reduction. Therefore, in this region, the anomalous co-deposition occurs. According to the widely accepted theory, Zn^2+^ has significantly faster electrodeposition kinetics than Co^2+^, and, thus, it deposits preferentially, resulting in a higher Zn percentage in the alloy [31,33,34]. Interestingly, the current density in this region is higher regardless of the ceria source, as the Ce species are electrochemically inert particles that usually suppress the rate of electrochemical reactions due to surface blocking [35]. A depolarization effect of ceramic particles was observed earlier, for example, in the electrodeposition of Co–Ni-Al_2_O_3_ [36] or Ni-SiC composites [37]. The most likely explanation for this anomaly is that some amount of metal ions is adsorbed at the oxide particles, and their mass transport is enhanced as compared to the diffusion of non-adsorbed metal ions [36]. The other plausible explanation can be the fact that the freshly deposited composite coating is of higher roughness as compared to the fresh Zn-Co deposit obtained during the polarization; it has a higher surface area, and, thus, a higher current is measured.

With a shift in the electrode potential to more negative values, hydrogen bubbles are observed, caused by hydrogen proton reduction. Consequently, the metal hydroxides are formed at a high pH value in the nearby substrate layer, blocking the electroactive surface and inducing the appearance of a current peak in region C [38]. A further potential sweep creates more bubbles that remove the blocking hydroxide, so the current continues with the undisturbed increase. The electrodeposition in region C was not in the focus of this work due to the parasitic hydrogen evolution.

#### 3.2.2. Composition and Morphology of Zn-Co-CeO_2_ Coatings Deposited under Magnetic Stirring

**Figure 4:**
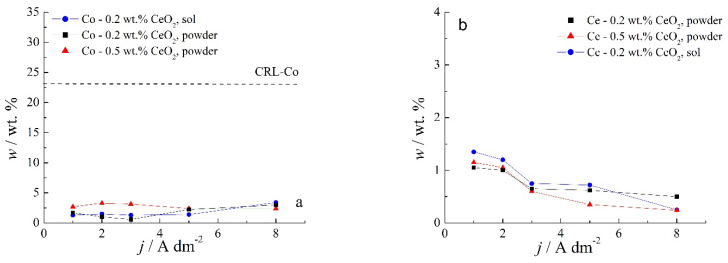
Dependence of the (**a**) Co and (**b**) Ce contents in Zn-Co-CeO_2_ coatings on the deposition current density for the coatings deposited from the magnetically stirred solutions with different sources and amounts of CeO_2_ particles.

Chemical content of composite coatings deposited from solutions containing different amounts of CeO_2_ powder, as well as with 0.2 wt.% sol, as a function of the deposition current density are shown in Figure 4.

**Figure 5:**
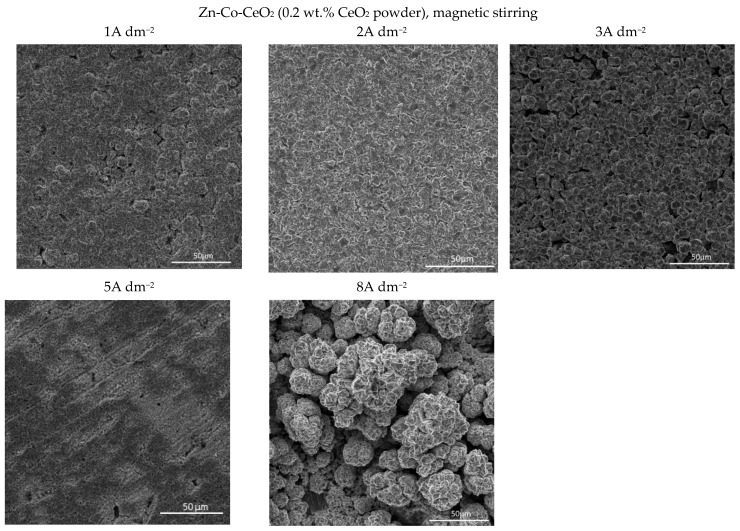
Morphology of the Zn-Co-CeO_2_ composite coatings deposited at different current densities from plating solution with 0.2 wt.% ceria powder.

**Figure 6:**
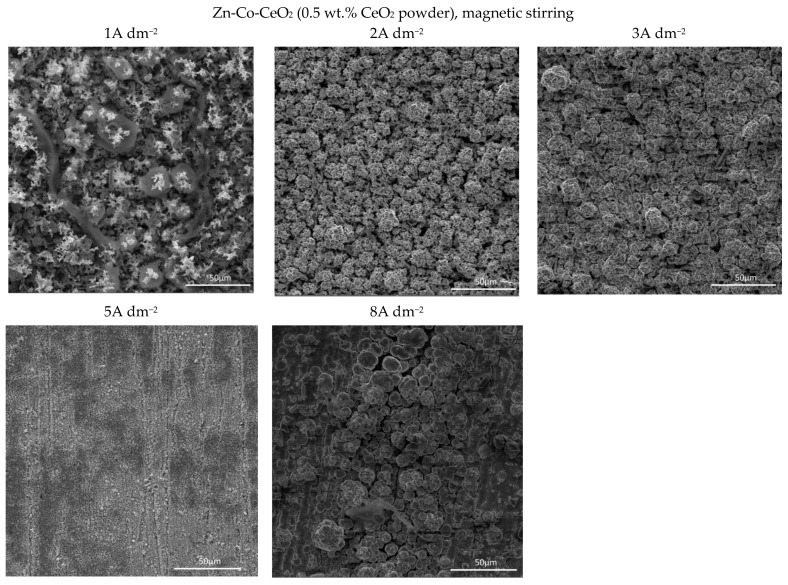
Morphology of the Zn-Co-CeO_2_ composite coatings deposited at different current densities from the plating solution with 0.5 wt.% ceria powder.

**Figure 7:**
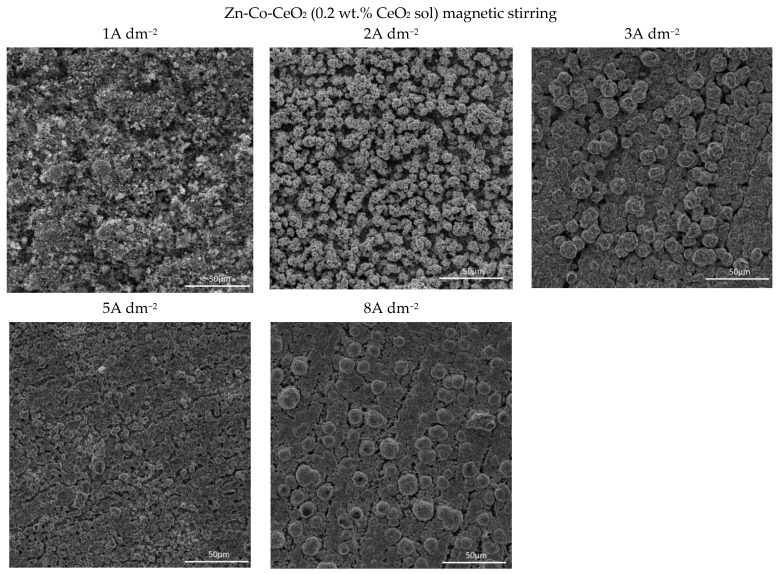
Morphology of the Zn-Co-CeO_2_ composite coatings deposited at different current densities from the plating solution with 0.2 wt.% ceria sol.

Several features could be inferred from Figure 4. The amounts of both Co and Ce are small, as expected based on the low contents of the CeO_2_ and Co ions in the plating solution. Still, a slight positive trend in Co content at a higher deposition current density could be observed for all plating solutions. Concurrently (Figure 4b), the rise in the deposition current density resulted in a decrease in the Ce content in the deposit, which is not an unusual phenomenon in the electrodeposition of composite coatings [18,39]. The smaller current densities 1 and 2 A dm^−2^ were beneficial for the particle content. Increasing particle concentration in the plating bath (0.5 wt.% CeO_2_ powder) did not cause arise in particle content in the deposit. However, home-made ceria sol favored the incorporation of particles in the Zn-Co matrix. The increase in current density caused the increase in the electrodeposition rate, whereas particles, mainly transported toward the cathode by agitation of the solution, did not have enough time to get trapped in the growing layer. The change in the concentration of the ceria powder in the plating solution from 0.2 to 0.5 wt.% was not beneficial to particle co-deposition due to the agglomeration of ceria particles [20], as large agglomerates tend to collide with the cathode surface or need a higher residence time to be incorporated into the growing layer. The slight rise in cerium content in the composite coating when ceria sol was used can be related to the smaller size of the particles that are uniformly distributed in the plating solution; therefore, the greater number of particles could have been trapped by the growing metallic matrix. Still, all composite coatings deposited anomalously since the Co content in the coatings was considerably below the compositional reference line (CRL), defined by Brenner [34] as the ratio of Co^2+^ to the total amount of metal cations (Co^2+^ and Zn^2+^) in the plating solution. The morphology of the electrodeposited composite coatings is shown in Figure 5, Figure 6 and Figure 7.

Quite a compact and uniform morphology was obtained by deposition at small current densities (1 and 2 A dm^−2^) from the solution containing 0.2 wt.% CeO_2_ powder. Small voids are present in 1 A dm^−2^ but lower than 3 A dm^−2^. A further rise of the current density to 5 A dm^−2^ resulted in an inhomogeneous surface, while the dendritic structure began to form at 8 A dm^−2^. It is also evident that the smooth and compact surface morphology obtained at smaller current densities became coarser with nodular patterning upon increasing the deposition current density. The size of the nodular structures shifted sharply from very few (around 3µm at 3 A dm^−2^) to approximately 40 µm agglomerates at the current density to 8 A dm^−2^. This kind of coating growth at 8 A dm^−2^ could be the consequence of the non-uniform distribution of the electric field. When any protuberance is formed on the growing surface, the ions are preferentially reduced on such nodules due to the “tip discharge effect” [40,41], resulting in larger nodules during advancement of the deposition time.

The increase in bath loading resulted in deterioration of the surface compactness and homogeneity. The surface appearance of these coatings (Figure 6) was changed as compared to those observed at a lower amount of particles (Figure 5). The morphology of the composite coating obtained at the smallest current density examined was quite heterogeneous. Irregularly distributed large zinc pallets surrounded by smaller snowflake-like structures were visible on the coating surface. The shift in current density to 2 and 3 A dm^−2^ resulted in a more homogeneous surface finish compared to 1 A dm^−2^, although with clearly visible porosity. The further shift in current density to 5 A dm^−2^ resulted in grain size refinement. The similarity in appearance of this coating with the morphology of the composite coating deposited under the same current density, but with lower bath loading, is obvious (Figure 5). The furrows of voids are visible in both composite coatings deposited at 5 A dm^−2^ (Figure 5 and Figure 6) probably as a result of an intense gas evolution from the surface, pointing out that at 5 A dm^−2^, the hydrogen evolution is significant during the electrodeposition process. The nodular structures are much bigger, appearing even on the coatings deposited at smaller current densities (2 A dm^−2^) when the bath loading is increased, which was not noticed for the coating morphology when a lower particle content was used (Figure 5). This kind of behavior suggests that the electric field was non-uniformly distributed during the electrodeposition from the solution containing 0.5 wt.% ceria powder.

The use of ceria sol resulted in grain size refinement in composite coatings (Figure 7). Due to the smaller size and the more uniform distribution of the sol particles in the plating solution, the morphology of the coating was improved. The electrodeposition at 2, 3, and 5 A dm^−2^ resulted in the significantly better appearance of the composite coating (the most homogeneous morphology). Even the increase in current density to 8 A dm^−2^ did not result in the formation of irregular large shapes and dendrites, such as in the case of the same concentration of ceria powder (Figure 5). However, the channel-like cracks are still observed in the samples deposited at 8 A dm^−2^ as the result of gas evolution as explained above.

#### 3.2.3. Corrosion Resistance of Zn-Co-CeO_2_ Coatings Deposited under Magnetic Stirring

**Figure 8:**
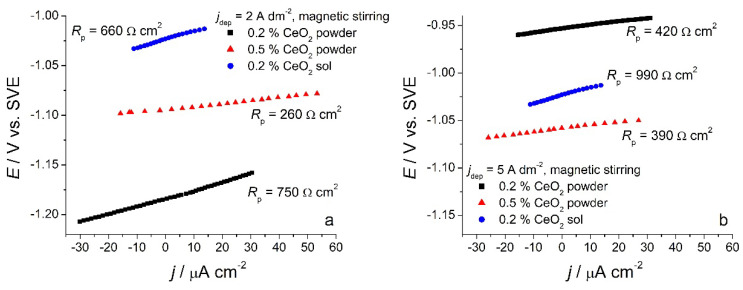
Polarization curves of different composite coatings deposited at (**a**) 2 A dm^−2^ and (**b**) 5 A dm^−2^ in the small range vs. *E*_ocp_ in 0.5 mol dm^−3^ NaCl.

Linear polarization measurements, as a fast method for determining polarization resistance, were used for preliminary tests of corrosion behavior of composite coatings. Samples deposited under magnetic stirring from the solutions containing 0.2 wt.% or 0.5 wt.% CeO_2_ powder were analyzed, as well as the ones deposited from the solution containing 0.2 wt.% CeO_2_ sol. Polarization resistance values (*R*_p_) were determined from the slope of the *dE*–*dj* curve (Figure 8) at the open circuit potential, and the results are presented in Table 1.

The smallest polarization resistance values were obtained for the composite samples deposited from the solution with 0.5 wt.% CeO_2_ powder for all deposition current densities. The lowest cerium content (Figure 4) and a rather non-homogenous morphology were responsible for unsatisfactory protection. On the other hand, the incorporation of Ce originating from the sol seems to be beneficial to the corrosion stability of Zn-Co-CeO_2_ composite coatings. This type of composite coating exhibited polarization resistance two times higher than that of the composite coating deposited from the plating solution containing 0.5 wt.% ceria. Finally, the polarization resistance values for the deposit obtained from the solution containing a lower concentration of ceria powder (0.2 wt.%) decreased significantly when the deposition current density was higher than 3 A dm^−2^. This is due to the low content of particles, which is also considerably lower when the current density increases, as well as to the low compactness of the coating. The higher ceria content, obtained at lower current densities (1 and 2 A dm^−2^), resembles the nanofillers by filling the eventual defects in the deposit and consequently improving barrier properties, thus suppressing the electrolyte uptake. In addition, cerium ions possess the ability to form a layer with a lower dissolution rate on a damaged metal substrate. The ceria precipitations along with barrier properties could slow down the advancement of corrosion [23].

### 3.3. Composition and Morphology of the Zn-Co-CeO_2_ Coatings Deposited under Ultrasound

**Figure 9:**
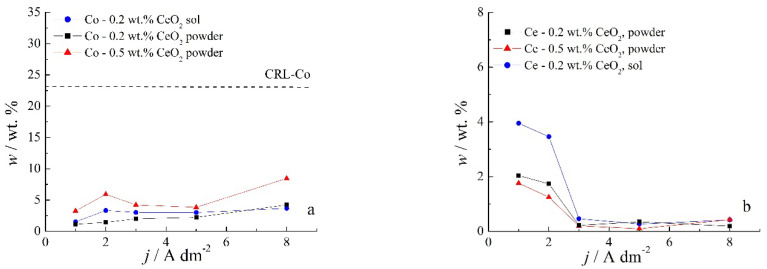
Dependence of the (**a**) Co and (**b**) Ce contents in Zn-Co-CeO_2_ coatings on the deposition current density for coatings deposited under ultrasound from the solutions with different sources and amounts of CeO_2_ particles.

Since agitation plays an important role in preventing particle agglomeration, deposition under ultrasound was further analyzed. The chemical content of composite coatings deposited at different current densities in an ultrasound bath from solutions containing different amounts of CeO_2_ from both sources is shown in Figure 9. A notably smaller amount of Co is incorporated with 0.2 wt.% CeO_2_ powder content in the plating solution than from the one containing 0.5 wt.% CeO_2_ powder, while sol-derived coatings gave Co content roughly in between these two. A somewhat greater Co content could be incorporated by US-assisted deposition as compared to agitation with magnetic stirring (Figure 4a). The anomalous Co co-deposition of Co with Zn could also be concluded for coatings deposited with US stirring, as plating solutions were of the same composition and small amounts of Co were determined.

The type of electrolyte stirring seems to have a pronounced influence on the particle incorporation. Coatings deposited under ultrasound agitation at lower current densities incorporated double the amount of Ce with respect to those obtained under magnetic stirring. However, the Ce content declined at 3 A dm^−2^ and remained at a plateau for higher deposition current densities. This trend was observed for all plating solutions, regardless of the ceria source. The particles are moved through the solution under the effect of ultrasound and gravitation force and also by electronic force due to the adsorbed cations on the particle surface [22], and, after reaching the cathode surface, they are entrapped by the growing layer. When the deposition current density was lower than 3 A dm^−2^, the transfer rate of the particles was higher compared to the growth rate of the deposit, resulting in a higher content of incorporated particles. When current density increases, the metal ions are moved faster compared to the particles, and the growth rate of the coating is higher; thus, particles do not have time to be captured by the growing layer. As a consequence, the content of the incorporated particles, i.e., cerium content, decreased by increasing the deposition current density (Figure 9b).

**Figure 10:**
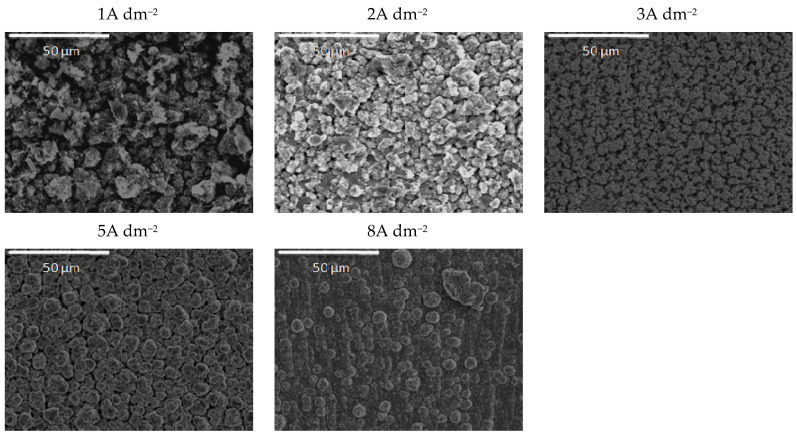
Morphology of the Zn-Co-CeO_2_ composite coatings ultrasounddeposited at different current densities from the plating solution with 0.2 wt.% ceria powder.

**Figure 11:**
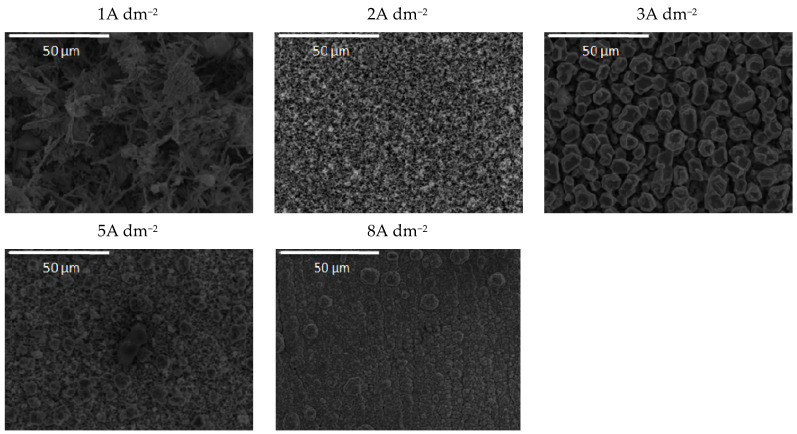
Morphology of the Zn-Co-CeO_2_ composite coatings ultrasounddeposited at different current densities from the plating solution with 0.5 wt.% ceria powder.

**Figure 12:**
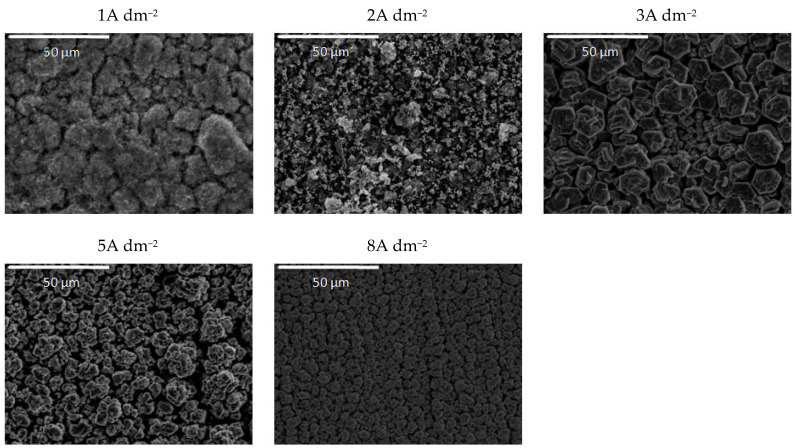
Morphology of the Zn-Co-CeO_2_ composite coatings ultrasounddeposited at different current densities from plating solution with 0.2 wt.% ceria sol.

The morphology of the ultrasound-deposited Zn-Co-CeO_2_ composite coatings is presented in Figure 10, Figure 11 and Figure 12. The dependence of the coating morphology on the deposition current density, as well as the particle source, is undoubted when ultrasound-assisted deposition is applied. A similar trend was observed for coatings deposited under magnetic stirring. The rise in deposition current density resulted in nodular-like patterning. The grain size was smaller when ultrasound was applied, as compared to magnetic stirring (Figure 5) even when a higher deposition current density was applied (8 A dm^−2^). When an ultrasound field is established in the plating electrolyte, various cavitation phenomena are triggered (e.g., micro-jetting, acoustic streaming, shock waves, etc.), enhancing the mass transfer and cathode current efficiency during the electrodeposition process [42]. As a result of the established cavitation effects in the plating solution, the surface of the cathode is cleaned by hydrogen bubbles, leading to a more compact coating. The existence of micro-turbulence in the solution, under the applied ultrasound, induces the de-agglomeration process of ceria powder particles and suppresses particle re-aggregation, as shown by PSD measurement (Figure 1). The grain size is reduced due to the influence of the abovementioned cavitation phenomenon. The applied ultrasound waves induce vesicles that hit the cathode surface and modify it, and, as a consequence, the number of active sites on the cathode grows, leading to faster nucleation and a smaller grain size of the deposit [43]. In addition, when using ultrasound during the electrodeposition, both the metal ions and ceria in the solution are transported faster to the electrode, and nanometric particles are easily and uniformly adsorbed onto the growing crystals, explaining the higher amount of incorporated particles (Figure 9b) compared to magnetic stirring (Figure 4). The faster mass transfer is also responsible for the fact that dendritic growth of the coatings does not occur even at a current density of 8 A dm^−2^, as was the case in the bath with magnetic stirring.

The addition of a higher amount of ceria powder into the plating solution (0.5 wt.%) together with the ultrasound at low current densities (1A dm^−2^) resulted in the snowflake-like structure of the deposit (Figure 11). The grain size of this coating was also reduced, and compactness was improved using ultrasound, as compared to those obtained by magnetic stirring (Figure 6). Finally, the ultrasound applied during the electrodeposition from the sol-containing bath (Figure 12) resulted in the formation of zinc hexagonal pallets of different sizes when the deposition current density was larger than 2A dm^−2^. The grain refinement for these coatings compared to the ones deposited under magnetic stirring (Figure 7) was most noticeable at 2 A dm^−2^. The coatings deposited at current densities in the range of 2–8 A dm^−2^ were smooth and compact, containing agglomerates of quite a uniform size.

**Figure 13:**
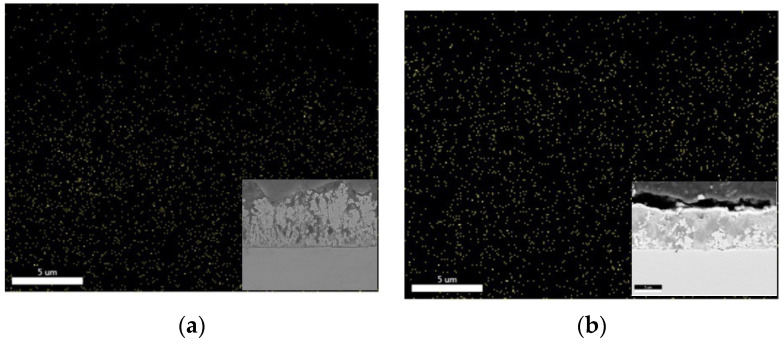
EDAX mapping of cerium in Zn-Co-CeO_2_ coatings deposited under ultrasound at 2 A dm^−2^ from solutions containing (**a**) 0.2 wt.% CeO_2_ powder and (**b**) 0.2 wt.% CeO_2_ sol, all elements. Cross-section SEM images are shown as inserts.

The distribution of Ce particles was quite homogenous, as shown in Figure 13 by the cross-section EDS mapping for samples deposited under ultrasound from two different solutions: 0.2 wt.% CeO_2_ powder (Figure 13a) and 0.2 wt.% CeO_2_ sol (Figure 13b) deposited at 2 A dm^−2^. The greater amount of Ce particles, homogenously distributed, could be observed when ceria sol was used as the particle source, which is in agreement with the results shown in Figure 9b.

### 3.4. Corrosion Resistance of Zn-Co-CeO_2_ Coatings Deposited under Ultrasound

The polarization resistance values (*R*_p_) of Zn-Co-CeO_2_ coatings deposited under ultrasound and different ceria sources are presented in Table 2. It can be seen that the utilization of ceria sol together with ultrasound agitation had a positive influence on the corrosion resistance of the Zn-Co-CeO_2_ coatings. Namely, the coatings obtained from the sol-containing solution show significantly higher resistance as compared to those obtained from the powder-containing baths. The polarization resistance values of the samples deposited from the sol bath were >1 kΩ cm^2^ for the deposition current density values up to 3 A dm^−2^. A further increase in the deposition current density resulted in a decrease in polarization resistance values, which is related to the reduction of cerium content in the coating and also to the deterioration of the surface homogeneity. Compact morphology and homogeneous cerium distribution and content in the coatings were found to be crucial for corrosion properties of Zn-Co-CeO_2_ composite coatings.

**Figure 14:**
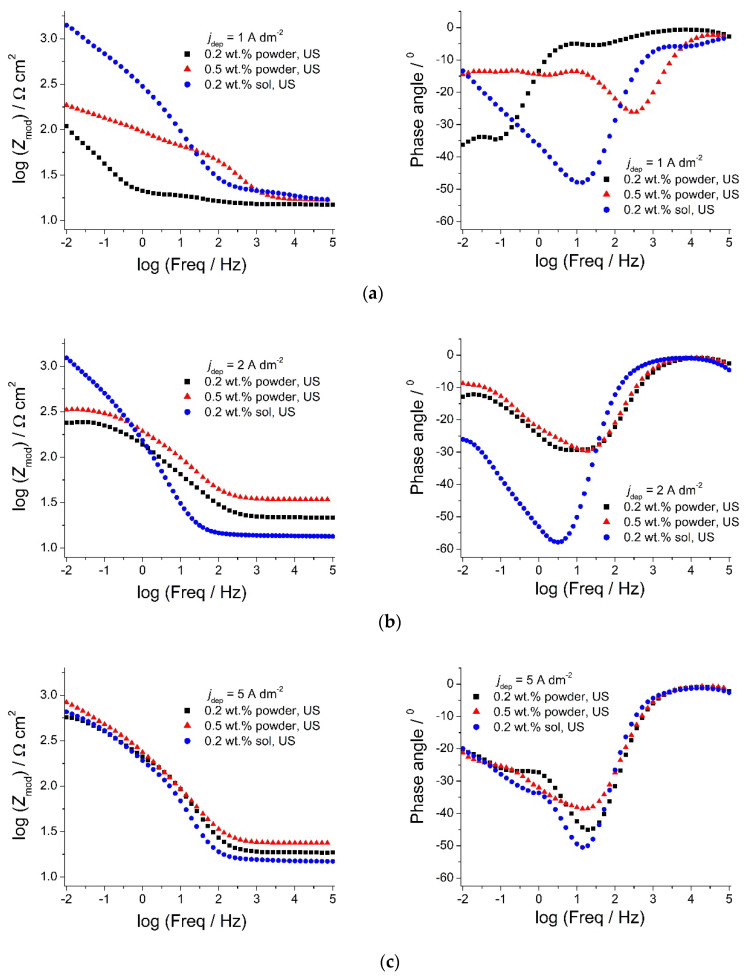
Bode plots of Zn-Co-CeO_2_ composite coatings deposited under ultrasound: influence of different deposition current densities: (**a**) 1 A dm^−2^, (**b**) 2 A dm^−2^, and (**c**) 5 A dm^−2^ and ceria sources on corrosion properties.

In order to achieve better insights into the effect of the type of ceria source used on the anti-corrosion properties of the coatings, electrochemical impedance spectroscopy measurements were performed in 0.5 M NaCl, 90 min after immersion in the corrosive medium. The corresponding Bode plots are presented in Figure 14.

From Figure 14a,b, it can be stated that *Z*_mod_ values for the composite coatings deposited from the solution containing ceria sol were much higher compared to the composites deposited from ceria powder when deposited at 1 and 2 A dm^−2^, suggesting better anti-corrosion properties. The phase angle of the sol-derived coating at low frequencies shifted toward higher values when shifting the deposition current density from 1 (Figure 14a) to 2 A dm^−2^ (Figure 14b), and this shape of spectrum is assigned in the literature to a ”blocking electrode’’ electrochemical response, where the electrical circuit has no current flow when the DC limit is achieved [44]. The *Z*_mod_ values at low frequency remain stable with the rise in deposition current density (Figure 14c). A reason for the improved anti-corrosion properties of ceria sol-derived composite coatings could be the higher amount of well-distributed and incorporated ceria particles. Besides being good corrosion inhibitors, ceria particles also enhance the barrier properties [44] of the coating, thus suppressing ingress of the electrolyte and access to the steel substrate. The agglomeration of particles is much more represented when commercial powder is used. Thus, large-size aggregates of particles present in the powder-containing plating solution were incorporated inside the coating, resulting in deterioration in corrosion resistance. The smaller *Z*_mod_ values (Figure 14a,b) with higher amounts of cerium (1 and 2 A dm^−2^) in the case of the composite coating deposited from the powder-containing solution, compared to the same coating deposited at a 5 A dm^−2^ current density (Figure 14c), is a consequence of its morphology.

**Figure 15:**
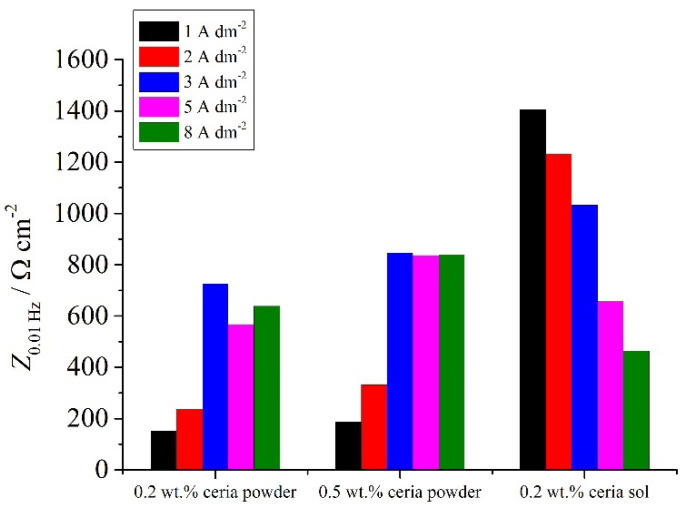
The low-frequency impedance modulus (at 0.01 Hz) for different samples deposited under ultrasound.

The impedance modulus values at low frequencies can be ascribed to processes occurring on the steel/electrolyte interface where corrosion occurs [45,46,47,48,49] and can be used as effective tool for determining the corrosion resistance properties of the system. The values of the low-frequency impedance modulus (Figure 15) of the Zn-Co-CeO_2_ and sol-derived coatings at 1–3 A dm^−2^ were higher than for the powder-containing particles. It is worth mentioning that the Z_0.01Hz_ values for Zn-Co alloys were around 114 Ω cm^−2^ for Zn-Co deposited under magnetic stirring and 150 Ω cm^−2^ for the alloy obtained under ultrasound power.

Based on all the presented results, it can be concluded that ceria particles incorporated into a Zn-Co matrix could be beneficial in the corrosion protection of steel, but only if they are homogeneously distributed in the plating solution and along the coating’s thickness. Protective properties are thus determined by the interplay between the chemical content of the coating and its surface morphology.

## 4. Conclusions

Based on the presented results, the following conclusions could be drawn:

- The Zn-Co-CeO_2_ composite coatings were successfully deposited from two plating solutions with a different source of ceria particles: ceria sol and ceria powder.
- The electrolytic bath exhibited greater stability when ceria sol was used, proven by the PSD measurements and sedimentation behavior. The agitation mode had a pronounced effect on stability, with ultrasound more effective in comparison to magnetic stirring.
- The polarization curves showed that Zn-Co deposition is anomalous in the presence of ceria particles, and the particles induce a small depolarization effect.
- With the increase in deposition current density over 2 A dm^−2^, the particle content in deposits decrease in the case of both examined ceria sources. The utilization of ultrasound instead of magnetic stirring resulted in a higher particle incorporation rate. Deposition at high current densities resulted in the formation of column-like structures.
- Ultrasound-assisted electrodeposition at small current densities was favorable for obtaining composite coatings with enhanced corrosion stability. The protection was more effective when ceria sol was used as a particle source, as revealed by the higher polarization resistance and greater low-frequency impedance modulus values for sol-derived composite coatings deposited under US at small current densities of 1–3 A dm^−2^.
- The protective properties of the composite coatings are determined by the interplay between the chemical content of the coating and its surface morphology.
- A complete understanding of the protection mechanism andthe role of ceria needs further study that would include the monitoring of the corrosion stability over a longer exposure time in a corrosive media.

## Figures and Tables

**Table 1:** Polarization resistance values of composite coatings deposited from solutions containing ceria powder (0.2 wt.% and 0.5 wt.%) and ceria sol (0.2 wt.%) under magnetic stirring.

*j*_dep_/ (A dm^−2^)	Zn-Co-CeO_2_ (0.2 wt.% Powder) /Ω cm^2^	Zn-Co-CeO_2_ (0.5 wt.% Powder)/ Ω cm^2^	Zn-Co-CeO_2_ (0.2 wt.% sol)/ Ω cm^2^
1	682	237	680
2	750	260	660
3	616	464	750
5	420	390	990
8	380	483	730

**Table 2:** Polarization resistance values of composite coatings deposited from the solutions containing ceria powder (0.2 wt.% and 0.5 wt.%) and ceria sol (0.2 wt.%) under ultrasound.

*j*_dep_/ (A dm^−2^)	Zn-Co-CeO_2_ (0.2 wt.% Powder)/ Ω cm^2^	Zn-Co-CeO_2_ (0.5 wt.% Powder)/ Ω cm^2^	Zn-Co-CeO_2_ (0.2 wt.% sol)/ Ω cm^2^
1	171	191	1220
2	331	400	1223
3	527	650	1050
5	590	660	713
8	570	630	698

## Data Availability

The data presented in this study are available on request from the corresponding author or co-authors. The data are not publicly available.

## Sample Availability

Samples are available from the authors.
